# Challenges of human resources management in nursing in Iran: A qualitative content analysis

**DOI:** 10.1002/nop2.393

**Published:** 2019-10-04

**Authors:** Vahid Zamanzadeh, Leila Valizadeh, Hanieh Neshat

**Affiliations:** ^1^ Department of Medical‐Surgical Nursing School of Nursing and Midwifery Tabriz University of Medical Sciences Tabriz Iran; ^2^ Department of Child and Family Health School of Nursing and Midwifery Tabriz University of Medical Sciences Tabriz Iran

**Keywords:** challenges, content analysis, human resource management, nurses, nursing

## Abstract

**Aim:**

Health service providers are appreciated among the vital components in a society wherein nurses are also considered as the main human resources. Thus, examining the existing challenges in managing these human resources through taking correct measures can contribute to identifying the priorities and consequently influence the quality of services provided. This study aims to investigate the challenges of human resources management in nursing from the perspective of professionals in Iran.

**Design:**

Qualitative content analyses.

**Methods:**

Present study is carried out using purposive sampling conducted on 12 nurses involved in different professional nursing positions. To this end, the participants' perceptions, opinions, beliefs and attitudes were collected via two focus group sessions. Data were collected during Febuary and March 2018.

**Results:**

From the perspective of the professionals, the challenges could be observed in a wide variety of human resources management dimensions in nursing such as job analysis, recruitment, as well as development and retention of workforce.

## 1 INTRODUCTION

Human resources are taken into account as the main capital of an organization, so an organization is required to take several measures in line with its mission and realization of its goals and strategies in the domain of human resources. It should be noted that human resources management (HRM) is a specialized field of study that makes efforts to estimate needs and to boost individual and organizational goals via innovating and developing different programmes, policies and activities (Yaghobian, 2008).

In this respect, health‐related organizations are among the most important ones in current societies. Human resources are similarly considered as a central element in the domain of health (Diallo, Zurn, Gupta, & Dal Poz, 2003). Meanwhile, the major part of the health workforce is comprised of nurses. This group can also play an important role in providing direct patient care; therefore, it is one of the foundations of each health system (Mudihanselage & Chamaru, 2015). Accordingly, proper management of these human resources can have a significant role in promoting the performance of health‐related organizations.

In the international arena, the management of nursing staff faces major challenges. The World Health Organization (WHO) has also referred to nursing shortage as a global challenge and reported a redoubled demand for nurses in the coming years (Buchan & Calman, 2006). Studies have further suggested that inappropriate nursing HRM can lead to augmented risks of exhaustion and burnout, job dissatisfaction and tendency to quit the profession in nurses and thus exacerbate the workforce shortage (Mudihanselage & Chamaru, 2015; Shin, Park, & Bae, 2018). Each of these negative outcomes can consequently impose additional costs, reduce quality of care and result in patient dissatisfaction (Jones, 2004; Koy, Yunibhand, Angsuroch, & Fisher, 2017). In this regard, a lot of studies have been done on various parts of nursing HRM that has been conducted mainly in the Western world (Irwin, Bliss, & Poole, 2018; Koy et al., 2017; Liu & Aungsuroch, 2018; Murray, Sundin, & Cope, 2018; Schalk, Bijl, Halfens, Hollands, & Cummings, 2010).

Besides, there are studies in the domain of nursing in Iran wherein the researchers have shed light on some dimensions of nursing in terms of HRM such as nursing shortage (Ebadi & Khalili, 2014; Hoseini‐Esfidarjani & Negarandeh, 2017; Negarandeh, 2015), nursing development and improvement programmes (Mesbahi & Tofighi, 2011; Rahimaghaee, Ehsani, Dehghan Nayeri, & Salavati, 2015; Salehi & Emamzadeh, 2014), job satisfaction (Asghari, Khaleghdoust, Asgari, & Kazemnejad, 2017), exhaustion and burnout (Hosseini, Sedghi Goyaghaj, Alamadarloo, Farzadmehr, & Mousavi, 2017; Rad & Hassani, 2014; Ranjbar, Mojalli, & NamdarAreshtanab, 2017), as well as factors affecting nurse retention (Chamani, Mahmoodi, & Babamahmoodi, 2012; Rivaz, Ebadi, & Momennasab, 2018). Although these investigations have illustrated some of the existing issues, it seems that conducting a comprehensive study and examining relevant challenges from the perspective of individuals working in different positions of such a profession can be a major step in recognizing priorities and strengthening existing evidence in this domain. Therefore, the purpose of this study was to examine the challenges of HRM in nursing profession in a more comprehensive manner with a scientific and expert view.

## 2 METHOD

This study was conducted using a qualitative approach with a content analysis method. To this end, the participants were selected through purposive sampling method in a way that they met maximum diversity in terms of their work experience, academic degree and job position. These individuals included 12 nursing professors, managers and experts from headquarters, as well as clinical nurses who attended two focus group sessions and expressed their perceptions, opinions, beliefs and attitudes towards the existing challenges in HRM in nursing. The project was as fieldwork for PhD students that Research Ethics Committee approval was obtained from college (letter number: 5/d/108503).

Prior to the given sessions, the research objectives and methodology were explained to the participants. Then, written consent for participation in the study along with permission to record the interviews was obtained. Also, the necessary explanations were given on voluntary participation in the study and the point that the participants could withdraw to cooperate with the researcher at any time as they wished.

The process of data analysis was also conducted based on qualitative content analysis and the steps proposed by Graneheim and Lundman (2004). To this end, at first, the interviews were typed verbatim. Then, the transcribed interviews and the field notes were reviewed for several times. Subsequently, the words, sentences or paragraphs in the transcripts were considered as semantic units and named by specific codes. The codes were also compared with each other in terms of similarities and differences and then grouped as more abstract categories. The process of codification and categorization of the data into the major and minor themes was continuously discussed in the research team, so that they reached a consensus on the codification and the classification of the data.

Use of the sampling method with maximum variation in this study could support fitness or transferability of the findings to other investigations. To obtain the verifiability and accountability of the study, the research steps and procedure were also accurately recorded and reported to provide the possibility of auditing and follow‐up for others.

## 3 FINDINGS

From the data extracted from the participants in the present study, four categories of policy‐making, being value‐based, scientific competency and interest along with some related sub‐categories were illustrated in Table 1.

**Table 1:** Formation of sub‐categories and categories

Category	Sub‐category
Policy‐making	Provision of a valid data bank Standardization of workforce estimate Creating a common language for policy makers and clarifying duties
Being Value‐based	Finding efficient talents Teaching values Consistency between candidates' values and professional positions. Value based organization and work environment
Scientific competency	Nursing education Individual motivation Motivational environment for learning
Interest	Social attractiveness Favourable work environment Supportive atmosphere at work Justice Professional independence

### 3.1 Policy‐making

Of the main challenges was the lack of transparency in the existing conditions for effective policy‐making. In this respect, the participants believed that it was necessary to have adequate knowledge of the current situation to make optimal decisions for the development and improvement of HRM in the domain of nursing.

#### 3.1.1 Providing a valid data bank

In this regard, the study participants stated that there was no comprehensive, formal and accurate information on existing conditions. For example, 1st participant explained about the numbers of active beds: “There is no much good organizational chart in terms of hospital beds and there are even beds available in hospitals that have not been included in the given chart.” The participants further emphasized the presence of such a data bank and said that the existence of this data bank could empower a system to effectively plan for the future. In this regard, 2nd participant noted that:

“There is no worth statistics or a bright future in the domain of human resources. I think it is very good if we can conclude and calculate the demands and the needs in our country as well as the provincial ones and also operationalize all of them.”

#### 3.1.2 Standardization of workforce estimates

Failure to follow specific standards in the estimation of the required nursing workforce was one of the issues highlighted in this study. For instance, one of the participants drew attention to the non‐observance of the approved guidelines for workforce estimation and stated that:

“We do not take the effective factors into account in the estimates. We ourselves ratified the Productivity Improvement Act while the current workforce estimates by the Ministry is on the basis of working for 198 hours which is the same as that before the adoption of the given Act.” (3rd participant)

Likewise, 4th participant considered uncertainty and confusion about taking a particular procedure as a major factor in this case and acknowledged that: “We need to determine whether we make a workforce estimate based on what international accreditation wants or just on the basis of maintaining our domestic minimums.”

#### 3.1.3 Creating a common language for policy‐makers and clarifying duties

Difference of opinion and absence of a common language between those involved in Iran's health system was posed as another challenge in this study. In this regard, 2nd participant argued that:

“The Vice‐Chancellor's Offices have not yet concluded a common language. The Vice‐Chancellor's Office for Education is making the estimates for itself and the Vice‐Chancellor's Office for Treatment is doing so alone. They are looking from their own structural perspectives.”

Besides, the participants pointed to the establishment of a Vice‐Chancellor's Office for Nursing and acknowledged that the unavailability of a common language and the existing controversies had not been resolved despite the presence of these Vice‐Chancellor's Offices. In this regard, one of the participants said that:

“We should reach a common language and a joint mission in the Ministry. If we do not have such a language, it seems like flagging a dead horse by the Vice‐Chancellor's Office for Nursing… we have created the Vice‐Chancellor's Office for Nursing and need to know how much powerful it is. I think we must accept that the Vice‐Chancellor's Office for Nursing is fully‐fledged and it still has not been mandated as we expect.” (4th participant)

### 3.2 Being value‐based

From the perspective of the participants, the necessity of having personal and ethical standards tailored to the nursing profession was a very critical challenge addressed in this regard. So, the importance of this issue was emphasized in each of the reported sub‐categories.

#### 3.2.1 Finding efficient talents

A group of the participants believed that individuals registered in nursing profession needed to meet the necessary ethical and talent‐related standards. In this regard, 5th participant said that:

“I think only those individuals can start nursing who are endowed with strong spirit at work as well as enough interest not just because of having an academic degree or finding a job.

#### 3.2.2 Teaching values

Some participants also added that ethics and values of this profession were required to be taught to students during their studies. One of the participants raised this challenge in this way:

There are nursing graduates who do not even care for nursing and they are always questioning the whole system and its values. So, in my idea, we need to make workforce training and development value‐based.” (2nd participant)

#### 3.2.3 Consistency between candidates' nursing values and their professional positions

Non‐selection of nurses in terms of values and ethical standards during recruitment was another problem addressed. For example, 4th participant mentioned the easier management of eligible workforces as well as the ability to measure these criteria during recruitment and said that:

“If we bring people who are in harmony with the values of the profession, it will be easier to keep them. In some countries, there are precise checklists that measure the qualities a person needs to have as a good and successful nurse.”

#### 3.2.4 Value‐based organization and work environment

Although the participants called attention to the need for value‐based standards appropriate to the nursing profession, most of them agreed that the value‐based organizational structure could have a great impact on individuals' behaviours. In this respect, 1st participant explained that: “What paradigm can prove that I will not change after 6–7 years…. It is a human‐made structure and you have to build it”. He further added that: “Being value‐based cannot be met by its own, the management should reach it.”

Besides, the study participants believed that, in some cases, appropriate organizational feedback would not be given to talented nurses who had met the value standards. One of the participants said that: “The main problem for nurses in Iran was insufficient motivation to work and lack of appreciation to efficient nurses” (5th participant).

### 3.3 Scientific competencies

From the participants' perspective, scientific competency in nurses was a very important requirement for providing proper services. They described the following cases as challenges in this domain.

#### 3.3.1 Nursing education

Lack of adequate support for novice nurses in terms of transition from the student role to the nursing role was recognized as a factor affecting the inadequacy of nurses' competence. Participants also believed that there were no comprehensive and obligatory guidelines for training new nurses. In this respect, 4th participant pointed that:

“We have problems with training newly‐recruited workforce. We need to specify our own routines in training new entrants; for example, whether we want to do mentorship or preceptorship. I think we need to write and run the same protocol.”

Of the other issues raised in this case were in‐service training programmes that were recognized as ineffective from the participants' perspective. One of them said that:

“in‐service training has lots of problems based on the reviews we have made. This type of training is not useful enough and we need to take this issue seriously.” (3rd participant)

#### 3.3.2 Individual motivations

Individual motivation in nurses also was considered as an important factor in acquiring scientific core competencies. In this regard, 1st participant pointed to lack of motivation for studying books and participation in educational classes and added that:

After graduation, all nursing books go to the archives and whatever the supervisor feels necessary is provided in a classroom wherein the attendees feel sleepy and have a sense of compulsion and overtime.

Other participants simply highlighted having the administrative role of nurses as a factor in their lack of motivation for gaining knowledge: “Unfortunately, we were so engaged with the administrative work that we ignored our studies and capabilities” (9th participant).

#### 3.3.3 Motivational environment for learning

Most participants emphasized that motivational organizational environments were crucial for acquiring scientific core competencies by nurses. One of the participants considered nursing work environment deprived of this motivation and said that: “Nurses who receive education are completely influenced by the environment and forget all about the process although they might be well‐educated” (11th participant). Fourth participant also reiterated that: “When nurses cannot have the power of thinking and autonomy and whereas everything is administrative, these problems are likely to come up”. In this respect, 10th participant said that:

“The system does not demand a nurse to practice the knowledge they have acquired; the system just demands a nurse to be task‐oriented and to perform a series of tasks well.”

### 3.4 Interest

The attractiveness of nursing at the community and the desirability of the workplace for nurses were the challenges detected in this regard.

#### 3.4.1 Social attractiveness

The low social attractiveness of nursing profession and lack of adequate efforts made to promote it were among the other challenges addressed in this study. In this respect, 6th participant said that: “In developed countries, there are attempts to make nursing as a selective priority for individuals through creating interest in society”. Second participant also pointed to the issue of social status and added that: “Social status is of utmost important for creating attractiveness in this profession.”

#### 3.4.2 Favourable working environment

During the focus group session, the challenges to creating a positive and pleasant working environment for nurses were illustrated as follows.

##### Supportive atmosphere at work

Considering the support provided for nurses by managers, 12th participant said that:

“The nursing management should not be ignored. If I am a good manager, I will have a good relationship with the nursing staff and do not let job burnout to be much annoying for them.”

5th participant noted the need for nurses to support each other:

“The Vice‐Chancellor's Office for nursing and the clinical nurses, I mean, authorities with nursing degrees have not provided nurses with an identity and also have not supported them as desired.”

##### Justice

In this respect, the participants emphasized the use of a specific framework for employee promotion; for example, 5th participant said that:

“The system needs to have a framework and even guidelines for the selection and promotion of individuals who take different responsibilities. In my opinion, biased and random choices in this respect can reduce motivation among the staff.”

10th participant also said that: “Unfortunately, the framework for encouragement and punishment is not clear enough… I think, we definitely need meritocracy not bureaucracy.” Among the other issues raised as a challenge to the pivotal role of justice was the income gap which was considered as a factor affecting lack of motivation in nurses: “When you see inequity and differences in income in the Ministry of Health, Treatment and Medical Education; you feel really upset and discouraged” (9th participant).

##### Professional independence

According to the participants, professional independence was a major challenge in Iran's nursing system. They also considered this issue as an important factor preventing a positive nursing work environment. In this respect, 11th participant considered asking about nurses' perceptions, opinions and beliefs as an important motivational factor in this case and said that: “They do not let us to give our comments, even in trivial issues. So, our self‐confidence can be hurt. They just view us as agents to implement theirs orders.”

##### Imposed activities

The participants believed that one of the problems facing nurses in some cases was that they were overwhelmed by others' duties that had not been properly met and also the nurses were required to correct them because they could affect the quality of nursing care. For example, 12th participant said that:

“Of the problems we are currently facing is that a nurse should assume lots of responsibilities for all relevant and unrelated jobs… If there is workforce shortage or no ability to do the tasks in an optimal way, this pressure is unconsciously imposed on the nurses from the laboratory technicians to the service staff, physicians, pharmacists and everyone.”

## 4 DISCUSSION

According to the results of the present study, the challenges faced by professionals in the HRM in nursing included the 12 sub‐categories which were consequently grouped into four main categories of policy‐making, being value‐based, scientific competency and interest.

The study participants considered the determining the valid data could determine the current status of Iran at the global level and consequently contribute to logical drawing of the prospects of activities ahead. In addition, the distribution of nurses in different provinces could be better determined and it is helpful in defining the standards for each region and adopting proper policies to recruit native interns. In this regard, Crisp and Chen (2014) provided and compared accurate statistics on nursing workforce in six WHO regions and then offered suggestions on supply and preparation of workforce in different areas.

The estimation of the workforce required in the organization could be the first step in terms of absorbing workforce whose standardization was emphasized by the participants in this study. One of the key issues discussed in the focus group sessions was the lack of estimating workforce based on the amount of nursing care needed in different departments and for various patients. In this regard, the methods used to estimate workforce in Iran were found to be not localized and the necessary timing and measurement had not been carried out in Iran's organizational environment. Considering the standardization of nursing estimates, other studies had been also carried out emphasizing the need to calculate the time required for nursing care provision, localization, as well as standardization of nursing workforce (Heydari & Tabari, 2015; Tabatabaee, Nekoie‐Moghadam, Vafaee‐Najar, & Amiresmaili, 2016; Tabatabaee, Vafaee‐Najar, Amiresmaili, & Nekoie‐Moghadam, 2017).

Because of dealing with human beings, nursing profession also requires specific values and ethical indicators, so having nursing‐related personal and ethical characteristics is a major global challenge that was mentioned by the participants in the present study. The use of nurses lacking motivation and interest can also reduce the effectiveness of the care system and cause irreparable harm to people's health. Callwood, Cooke, Bolger, Lemanska, and Allan (2018) also emphasized the need to measure such criteria in the admission process of nursing students and underscored its implementation in the United Kingdom. In 2016, the WHO further introduced providing education to qualified, motivated and responsible nurses as a new direction worldwide and considered it as an international strategy to strengthen the quality of healthcare services.

Another challenge raised in this study was the scientific competency of nurses. The participants pointed to the ineffective preparation of newly recruited nurses to start work as well as the inadequacy of in‐service training among the issues affecting the scientific competency of nurses. In a review study of 45 articles, Murray et al. (2018) concluded that there was uncertainty of sufficient knowledge among novice nurses about patient safety, so providing support for them during the transition from this stage seemed of utmost importance. Unfortunately, no training programme and standard guidelines have been still developed in Iran to prepare novice nurses to provide high‐quality services. In a meta‐review study, Reinhard highlighted the major benefits for these courses including increased professional knowledge of novice nurses (Reinhard, 2017). In current study, furthermore, in‐service training programmes were questioned in terms of their effectiveness by the professionals participating. Several studies have been also done assessing the quality of educational programmes for nurses in Iran via addressing the existing shortcomings and deficiencies and their causes in this domain (Rahimaghaee et al., 2015; Salehi & Emamzadeh, 2014).

Result of study showed that nursing should be introduced as an attractive profession at the community level and thus, it should be placed in the priority of the fields of study for candidates participating in the entrance exam of universities. On the other hand, increasing the social attractiveness of this profession at the community level could be a good incentive for the willingness to work among graduates of this profession. The low charm of the nursing work environment was also highlighted by the study participants. In this regard, they pointed to problems such as lack of supportive atmosphere at workplace, meritocracy and justice, work independence and overloaded tasks from others assigned to nurses as the factors reducing the attractiveness of nursing work environment. According to a report released by the International Council of Nurses in 2007, a positive working environment can lead to the retention of nurses in an organization, better teamwork, increased productivity, more continuity of patient care and finally promotion of patient outcomes (International Council, 2007). In this case, other studies have been also conducted and factors such as pays and benefits, management style, nature of work environment, autonomy and authority, participation in decision‐making, use of fair evaluation practices and individual development opportunities have been introduced as ones affecting the attractiveness of the workplace (Rivaz et al., 2018; Schalk et al., 2010; Smith, Hood, Waldman, & Smith, 2005).

## 5 CONCLUSION

The results of the present study shed light on the challenges of HRM in nursing. The findings stemmed from the perspectives of professionals could be also examined and discussed in the framework of a wide range of HRM dimensions such as job analysis, recruitment, as well as development and retention of workforce. Adopting policies, making proper decisions, maintaining and implementing professional values, enough scientific competency and popularity of the profession among nurses and within communities could have positive effects in terms of meeting the ever‐increasing needs in society to the nursing profession as well as training and retention of native experts. Since it seemed that top managers' opinions about nursing problems could promote the existing situation, it was better to ask about and make use of their views on the current challenges of nursing profession.

## CONFLICT OF INTEREST

There are none to declare.

## AUTHOR CONTRIBUTIONS

VZ: design the study, interpretation of data, and writing the paper; LV: performing the focus group and writing the paper; HN: collection, analyze and interpretation of data and writing the paper.
